# A Complex Presentation of Multiple Myeloma With Renal Complications in a Young Female: Diagnostic Challenges and Treatment Approach

**DOI:** 10.1002/jha2.1093

**Published:** 2025-01-24

**Authors:** Hem Prajapati, Yash M. Patel, Ajay C. Parmar, Sahaj Y. Patel, Tasin Mohammedyakub Shaikhjiwala

**Affiliations:** ^1^ Department of Medicine Government Medical College Baroda India; ^2^ Department of General Medicine S.S.G. Hospital and Medical College Baroda Vadodara India; ^3^ Department of Medicine Bukovinian State Medical University Chernivtsi Ukraine

**Keywords:** multiple myeloma, Renal Complication, Young Female

## Abstract

A previously healthy 30‐year‐old woman experienced worsening back pain, fatigue, weakness, loss of appetite, and facial puffiness. After 18 months of these symptoms, she was diagnosed with multiple myeloma, which was also damaging her kidneys. The treatment involved a combination of medications and blood transfusions, leading to improved kidney function. Despite the challenges of managing her condition as a mother of young children, she remains hopeful. This case emphasizes the need for awareness of multiple myeloma in younger patients and the importance of early diagnosis and multidisciplinary care for managing this chronic illness.

**Trial Registration**: The authors have confirmed clinical trial registration is not needed for this submission

## 1 Introduction

Multiple myeloma (MM) is the most common primary bone malignancy, accounting for 10% of all hematologic malignancies and 1% of all cancers [1]. It is generally a disease of the geriatric population, with the mean age at diagnosis between 65 and 68 years [2]. Approximately 3% of MM cases are identified in patients younger than 40 [3]. MM typically presents with anemia, back pain, and an elevated sedimentation rate, particularly in older men. Patients may experience rib pain and pathologic fractures, especially of the hip. Physical examination can reveal pallor, elevated temperature, bone tenderness, and soft tissue masses, with potential spinal cord compression causing neurological deficits. This malignancy of plasma cells leads to bone destruction, bone marrow replacement, and paraprotein formation, resulting in anemia, osteoporosis, hypercalcemia, and pathologic fractures over time.

In this case report, we describe a young female patient with a complex presentation of MM, including significant renal complications. This case highlights the need for a thorough diagnostic approach and the challenges associated with managing a disease typically seen in older adults. Given the rarity of MM in younger populations, this case provides insight into differential diagnosis and effective management strategies.

## 2 Case

A 30‐year‐old married female residing at Maneja, Vadodara. The patient was relatively asymptomatic before 1.5 years following which she developed backache, which was insidious in onset, gradually progressive, non‐radiating, dull aching, increased by body movements, relieved by medication, and not associated with the diurnal variation. Hair loss, which was gradually progressive, was not associated with itching or any skin lesions. Easy fatigability in the form of difficulty in doing day‐to‐day work increased during exertion. Generalized weakness which was gradually progressive, loss of appetite, breathlessness which was insidious in onset, gradually progressive, increased during exertion, not present during rest, NYHA grade 2, not associated with orthopnea and PND. Facial puffiness which was more in the morning. The patient was found hypertensive for 1 week.

## 3 Investigation

### 3.1 Lab Investigation

Patient Has low Hb, PCV and RBC with Raised ESR (Table 1). Shows High S. Urea and S. Creatinine and Low A/G Ratio (Table 2). Also has hypercalcemia and hyperphosphatemia (Table 3). Has Proteinuria (Table 4), Gamma band on UPEP (Table 5) and shows monoclonal band(Table 6).

**TABLE 1:** Shows anemia (low Hb, PCV, and RBC) and raised ESR.

Parameters	Result	Ref. range
**Hemoglobin**	**4.2 g/dL**	11–15 g/dl
**RBC**	**1.52 × 10^6^/cmm**	3.8–4.8 **×** 10^6^/cmm
**PCV**	**13.90%**	36%–46%
MCV	91.6 fL	83–101 fL
MCH	27.8 pg	27–32 pg
MCHC	30.4 g/dL	31.5–34.5 g/dL
**RDW**	**16.7%**	11.6%–14%
WBC	5210/cmm	4000–11,000/cmm
N/L/E/M	52/39/08/01	40%–80%/20%–40%/1%–6%/2%–10%
Platelets	329,000/cmm	150,000–410,000/cmm
**ESR**	**150 mm**	0–12 mm
RETIC	0.5%	0.5%–2.5%

**TABLE 2:** Shows renal damage (high S. urea and S. creatinine) also hyperproteinemia and low A/G ratio.

Parameters	Result	Ref. range
**S. urea**	**78 mg/dL**	14–40 mg/dL
**S. creatinine**	**2.78 mg/dL**	0.6–1.2 mg/dL
S. sodium	141 mEq/L	135–145mEq/L
S. potassium	3.7 mEq/L	3.5–5.1 mEq/L
S. total bilirubin	0.6 mg/dL	0.1–1.2 mg/dL
S. direct bilirubin	0.2 mg/dL	0–0.4 mg/dL
S. indirect bilirubin	0.4 mg/dL	0.1–0.8 mg/dL
S. ALT (SGPT)	7 IU/L	0–40 IU/L
S. AST (SGOT)	15 IU/L	0–37 IU/L
S. ALP	79 IU/L	64–306 IU/L
**S. total protien**	**9.90 g/dL**	6–8 g/dL
**S. albumin**	**2.20 g/dL**	3.2–5 g/dL
**S. globulin**	**7.70 g/dL**	2.3–3.6 g/dL
**S. A/G ratio**	**0.29**	1–2

**TABLE 3:** Shows hypercalcemia and hyperphosphatemia.

Parameters	Result	Ref. range
S. LDH	288	230–460 U/L
S. calcium	8.9 mg/dL	8.5–10.5 mg/dL
**S. ionised calcium**	**1.41 mmol/L**	1.12–1.32 mmol/L
S. uric acid	7.01	3.5–7.2 mg/dL
**S. PO4**	**6.32**	3.4–4.5 mg/dL
**S. chloride**	**112 mmol/L**	98–110 mmol/L

**TABLE 4:** Shows Proteinuria.

Parameters	Result	Ref. range
Color	Pale yellow	
pH	7.0	
Specific gravity	1.013	1.005–1.030
**Protien**	**2 (+) (Rechecked by SSA method)**	Nil
Glucose	Nil	Nil
Ketones	Nil	Nil
Bilirubin	Nil	Nil
Occult blood	1 (+)	Nil
Leucocytes	Nil	Nil
White (pus) cells	Occasional / hpf	0–5 / hpf
Red cells	1–2 / hpf	0–2 / hpf
Epithelial cells	Occasional / hpf	0–15 / hpf
Crystals	Not seen	
Casts	Not seen	
Yeast	Not seen	

**TABLE 5:** Shows gamma band on UPEP.

Parameters	Result	Ref. range
**24 h urinary protein**	**17.36 g/24 h**	< 0.2 g/24 h
**S. beta 2 microglobulin**	**29.86 mg/L**	0.81–2.19 mg/L
**Urine protein conc**.	**213 mg/dL**	0–15 mg/dL
**Urine protein/creatinine ratio (UPCR)**	**7693 mg/g creatinine**	0–300 mg/g creatinine
**Urine albumin**	**572 mcg/mL**	< 150 mg/g creatinine
**Urine albumin/creatinine ratio (UACR)**	**2067 mg/g creatinine**	< 30 mg/g creatinine
**Urine protein electrophoresis (UPEP)**	**Sharp band seen in gamma region**	

**TABLE 6:** Shows monoclonal band.

Parameters	Result	Ref. range
**S. total proteins**	**12.7 g/dL**	6.3–8.2 g/dL
**S. albumin**	**2.7 g/dL**	3.5–5.0 g/dL
S. alpha 1 globulin	0.56 g/dL	0.21–0.35 g/dL
S. alpha 2 globulin	1.09 g/dL	0.51–0.85 g/dL
S. beta 1 globulin	0.43 g/dL	0.34–0.52 g/dL
**S. beta 2 globulin**	**0.93 g/dL**	0.23–0.47 g/dL
**S. gamma globulin**	**7.29 g/dL**	0.8–1.35 g/dL
**Monoclonal band**	**Detected**	

### 3.2 Radiologic Image

**FIGURE 1:**
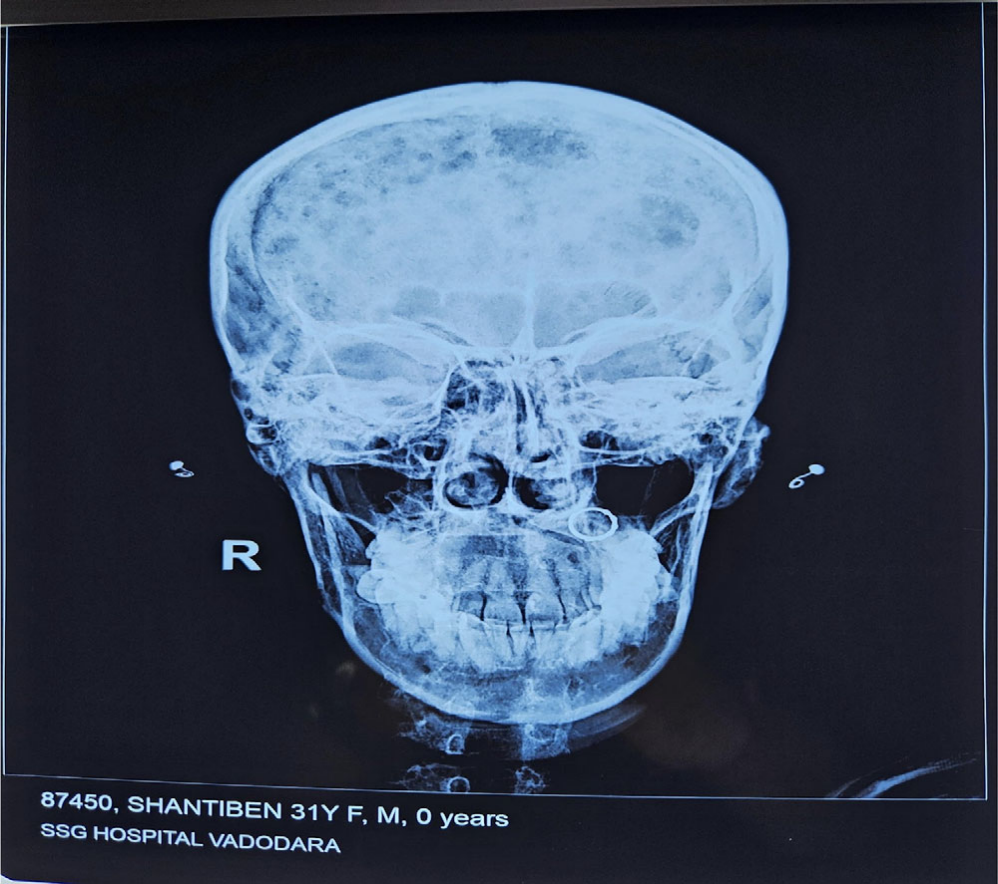
X‐ray of multiple lytic lesions in the skull in AP view.

**FIGURE 2:**
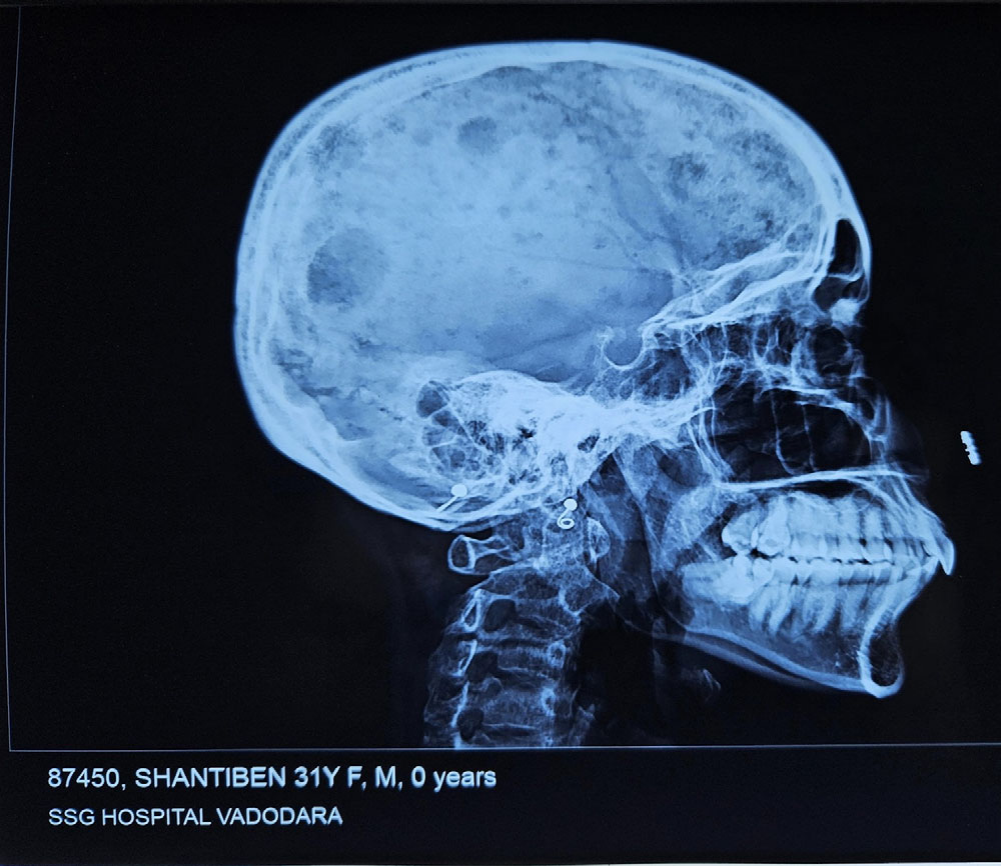
X‐ray of multiple lytic lesions in the skull in Lateral view.

**FIGURE 3:**
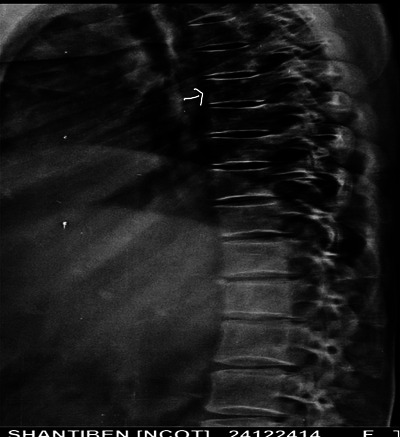
X‐ray of thoracic spine showing lytic lesion.

X‐Ray: Figures 1 and 2 show multiple lytic lesions in the skull. Also Figure 3 is showing the X‐ray of Thoracic spine having lytic lesion.

**FIGURE 4:**
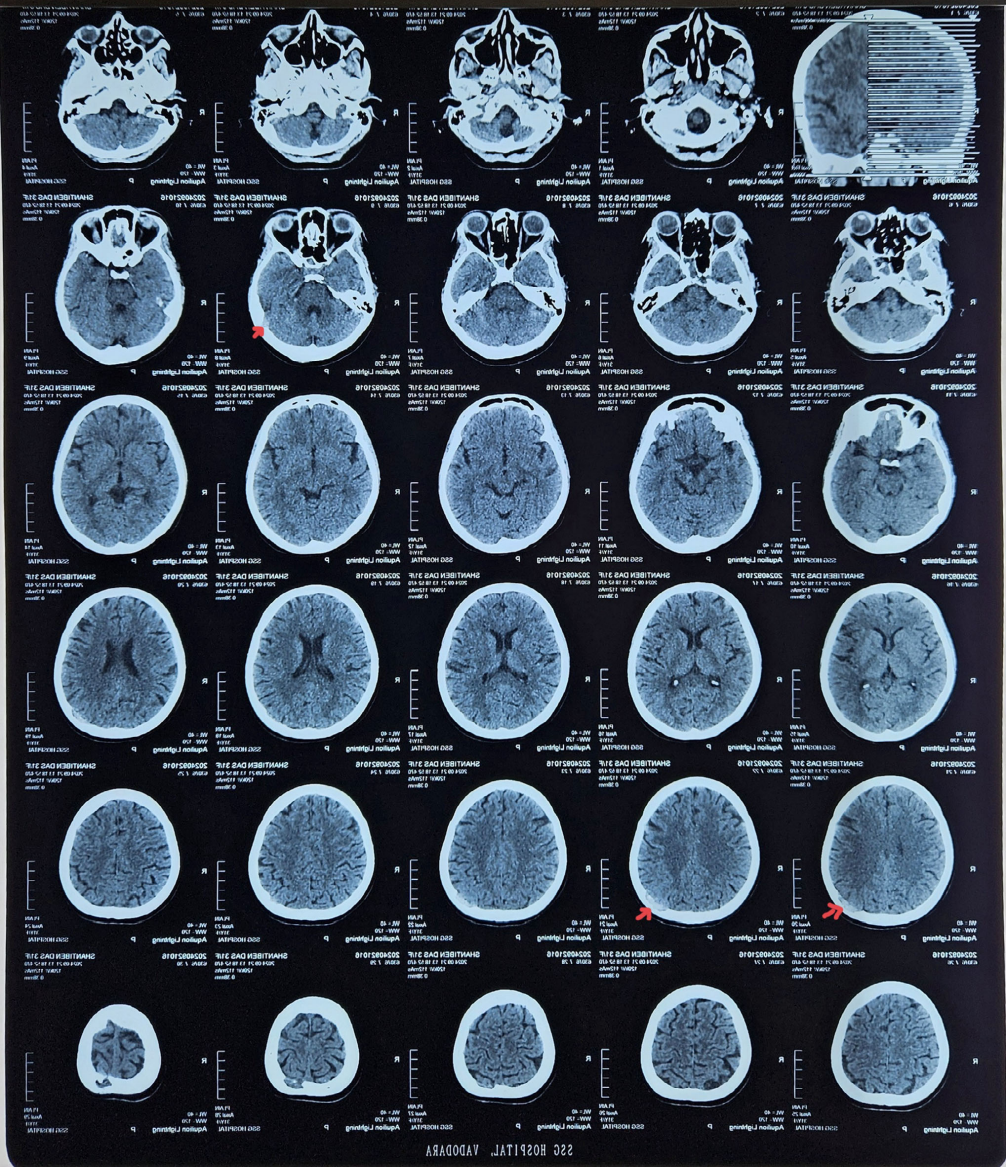
CT scan of head showing lytic lesion as indicated by the red arrow.

CT Head (Figure 4) Shows lytic lesion of skull USG abdomen:

· Liver, pancreas, and spleen—normal size and echopattern.
· Gall bladder—minimally distended.
· Kidneys—right—size: 110 × 42 mm, CMD preserved; left—size: 116 × 44 mm, CMD preserved.

B/L kidneys appear bulky and show raised cortical echogenicity.

### 3.3 Renal Biopsy

The renal tissue analysis reveals six glomeruli with no signs of global glomerulosclerosis. The glomeruli are enlarged with normal cellularity and structure. However, severe acute tubular injury (ATI) is evident, characterized by flattening, loss of brush borders, and reactive cell nuclei. Protein resorption droplets, casts, and mild tubular atrophy are present. The interstitium shows focal edema and lymphoid infiltrate, with no amyloid deposits detected. These findings indicate lambda‐restricted light‐chain nephropathy causing ATI and proximal tubulopathy.

### 3.4 Differential Diagnosis

A thorough differential diagnosis for a 30‐year‐old female points to MM, confirmed by her symptoms of back pain, weakness, renal impairment, anemia, hypercalcemia, and IgG‐lambda monoclonal gammopathy. Renal biopsy findings reveal light‐chain nephropathy, with bone marrow studies showing over 20% plasma cells. Chronic kidney disease (CKD) is another significant diagnosis, likely secondary to myeloma. Anemia of chronic disease is considered due to fatigue and pallor. While lupus nephritis and hyperparathyroidism are less likely, they remain possibilities. Hypertension should also be investigated, highlighting MM as the primary diagnosis alongside other contributing conditions.

### 3.5 Probable Diagnosis

–Multiple myeloma (IqG‐Л) (ISS Stage 3) with lambda‐restricted light‐chain nephropathy and tubulopathy.

### 3.6 Therapeutic Intervention

The patient was admitted to the hospital, and a multidisciplinary treatment plan was initiated:

· **Bortezomib (2 mg/week)**: Proteasome inhibitor targeting plasma cell apoptosis, crucial for both myeloma control and renal protection.
· **Lenalidomide (25 mg)**: Immunomodulatory agent used in combination to enhance anti‐myeloma effects.
· **Dexamethasone (40 mg/week)**: Corticosteroid for anti‐inflammatory and anti‐myeloma purposes.
· **Supportive therapies**: Packed cell volume (PCV) transfusion for anemia, antihypertensive drugs (furosemide, enalapril), and gastric protection (pantoprazole).

## 4 Follow‐Up and Outcomes

MM is an incurable chronic disease, especially in ISS Stage 3, which correlates with a poorer prognosis. However, early treatment with bortezomib and lenalidomide can improve survival and quality of life. Light‐chain nephropathy complicates the prognosis, necessitating ongoing monitoring of renal function and plasma cell levels.

## 5 Discussion

This case involves a 30‐year‐old woman with a complex and progressive set of symptoms affecting multiple systems over 1.5 years. Clinical findings, lab results, and a renal biopsy led to a diagnosis of MM with associated light‐chain nephropathy and tubulopathy. MM typically affects older adults, with the average age of diagnosis around 70 years [4]. Cases of MM in individuals under 30 are extremely rare, accounting for only 0.02% of all diagnosed cases [5], and only a handful of cases have been reported in this age group [6]. Interestingly, studies suggest that younger patients with MM often experience a more aggressive form of the disease, though they may also respond better to treatment [7].

MM is a cancer characterized by the abnormal growth of plasma cells originating from a single clone [8]. This leads to significant hypogammaglobulinemia, which is the primary reason for serious infections in patients who would otherwise have normal immune function. The exact cause of myeloma is still unclear, but various cytogenetic abnormalities have been linked to the disease. These abnormalities, identified through fluorescent in situ hybridization (FISH), commonly involve regions such as 1q21, 11q13, 14q32, 15q24, and 17p3, along with IGH rearrangements and TP53 deletions, or gains on chromosomes 9, 11, or 15. Specifically, the gain of 1q21 (CKS1B), as found in our patient, is associated with a poor prognosis [9, 10].

The diagnosis of MM requires either more than 10% plasma cells in the bone marrow with evidence of organ damage or over 60% plasma cells without organ damage. Signs of organ damage include CRAB symptoms: hypercalcemia, renal failure, anemia, and lytic bone lesions detected on imaging [11].

In MM, the plasma cells typically express CD38, CD56, and CD138. Overproduction of interleukin‐6 (IL‐6) is thought to be crucial for the survival of myeloma cells. Other factors like VEGF, TGF‐β, and the receptor activator of NF‐kB are also implicated in the disease [12]. The bone lesions in MM are believed to result from increased osteoclast activity and suppressed osteoblast activity, which is why serum alkaline phosphatase (ALP) levels often remain normal. The osteoclast activation is thought to be driven by an increase in RANKL on osteoblasts and a reduction in osteoprotegerin (OPG), leading to hypercalcemia [13].

Anemia occurs as myeloma cells infiltrate the bone marrow, disrupting normal blood cell production. Renal failure is caused by cast nephropathy, where light chains accumulate in the kidney tubules, leading to kidney dysfunction.

Severe episodes of infections are commonly seen in MM cases and are described as the leading cause of death in such patients. These cases are most often associated with bacterial infections of the lung and the urinary tract. Nonetheless, there are also reports of viral and fungal infections [14].

## 6 Key Clinical Message

MM, though typically diagnosed in older adults, can also be present in younger individuals with non‐specific symptoms like back pain, fatigue, and facial puffiness due to kidney involvement. Early diagnosis and treatment are crucial to managing both cancer progression and associated organ damage. This case highlights the importance of considering MM in younger patients with unexplained symptoms and the need for a multidisciplinary approach involving oncologists and nephrologists for the long‐term management of this chronic condition (Tables 1, 2, 3, 4, 5, 6).

## 7 Patient's Perspective

I always saw myself as healthy, so when I started experiencing back pain, fatigue, and facial puffiness, I didn't think much of it. After a year and a half, I saw a doctor and was shocked to be diagnosed with MM, a cancer that was also damaging my kidneys. The treatment was intense, involving strong medications and blood transfusions, but my kidney function improved, offering some hope. Although MM is a chronic condition requiring lifelong care, I'm focused on staying strong for my family and following my treatment plan, learning to pay attention to my body's signals.

## Conflicts of Interest

The authors declare no conflicts of interest.

## Data Availability

Data sharing not applicable to this article as no datasets were generated or analyzed during the current study.
